# Invasive procedures in nursing: advancing treatment outcomes with innovative approaches

**DOI:** 10.1590/1518-8345.0000.4533

**Published:** 2025-07-11

**Authors:** Sara Mogedano-Cruz, Carlos Romero-Morales

**Affiliations:** 1 Universidad Europea de Madrid, Facultad de Medicina, Salud y Deporte, Madrid, Spain.

**Figure:**
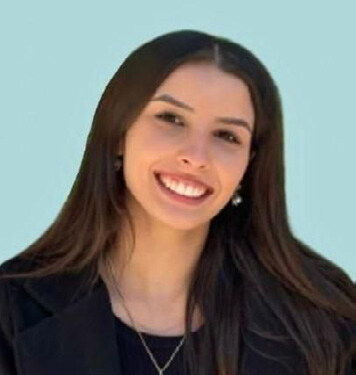

**Figure:**
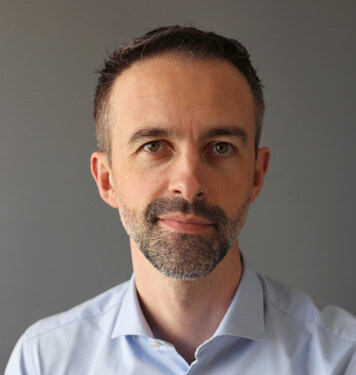

Invasive techniques in nursing are essential procedures that are performed across various healthcare settings, and they play a critical role in improving patient outcomes. The scope of practice for nurses varies by country, and it is important to understand the regulatory and educational frameworks that shape the role of nurses in performing these procedures^(1)^.

In Spain, nurses must undergo specialized education and training to perform these interventions safely. The training often includes both theoretical knowledge and practical experience, which is acquired through nursing programs, post-graduate courses, and clinical supervision. This training ensures that nurses are equipped with the skills necessary to perform invasive procedures effectively and safely. In addition to formal education, nurses must adhere to strict protocols and guidelines to minimize risks and complications.

Invasive techniques are defined as procedures that involve penetration into the body in order to treat diseases and improve the prognosis and quality of life of patients. In the increasingly complex healthcare environment, where patients often present multiple comorbidities, it is its critical that healthcare professionals receive adequate education to perform these interventions safety and effectively. While the frequency of such procedures may vary depending on the clinical context, many of these techniques are routine in hospitals and are performed on a daily basis^(2)^.

The relevance of this techniques lies in their ability to provide access to internal body areas, facilitating the administration of necessary treatments. These interventions are not only essential for acute care, but also play an important role in the management of several long-term conditions. Proper execution of these techniques can significantly improve patient outcomes, reduce the likelihood complications, and positively affect overall quality of life^(2-3)^.

One of the most common invasive techniques in nursing is venous access cannulation, a procedure used for the administration of fluids and medications, especially in emergency situations. Moreover, in recent years, peripheral central access cannulation has gained relevance in the management of patients requiring prolonged intravenous treatments. This technique allows for the administration of medications and fluids while avoiding damage to peripheral veins. The use of these therapeutic options has resulted in a positive impact on the hospital stay and patient satisfaction^(3)^.

Bladder catheterization is another fundamental technique used to drain urine from the bladder in patients with urinary retention or to accurately monitor renal function. Although it is a routine procedure, it carries significant risks, such as urinary tract infections. Therefore, implementing specific insertion and maintenance protocols has proven effective in reducing the incidence of adverse effects^(4)^.

Peripheral nerve stimulation has recently been integrated as a complementary approach in pain management, providing an innovative strategy for both acute and chronic pain. This technique not only helps to decrease pain intensity but also allows for a more precise assessment of nerve function. Research suggests that this type of stimulation may be effective in treating various painful conditions, thereby improving patients’ quality of life^(5)^.

Lumbar puncture is another relevant procedure used to obtain cerebrospinal fluid for diagnostic or therapeutic purposes. This intervention requires a deep anatomical understanding and specific skills to minimize the risk of complications^(1)^. Finally, abscess drainage is crucial in treating localized infections, preventing the progression of the infection and avoiding complications such as sepsis. Proper education to perform these procedures must encompass both technical aspects and aseptic management, as inadequate handling can increase the risk of additional complications^(2-4)^.

A thorough understanding and correct execution of these techniques not only enhance immediate patient care but also affect the long-term management of various conditions. Continuing education of healthcare personnel is essential to ensure the safety and effectiveness of these procedures, as well as to minimize complications and improve clinical outcomes. Education should be an ongoing process that adapts to the changing needs of the clinical environment and advancements in healthcare practice^(1-2)^.

Furthermore, a multidisciplinary approach to healthcare highlights the importance of collaboration among different professionals. Nurses, as primary care providers, must work together with doctors, physiotherapists, and other specialists to offer comprehensive care that addresses the physical, emotional, and social needs of patients. This collaboration not only improves the quality of care, but also contributes to creating a more positive patient experience, facilitating quicker recovery^(1)^.

It is important to note that the list of invasive procedures discussed in this article is not exhaustive, as there are many other procedures performed by nurses depending on the clinical context. The scope of practice for nurses varies internationally, and it is essential to acknowledge these differences when discussing invasive procedures in nursing practice.

In conclusion, invasive treatment techniques are indispensable components in nursing practice and patient care. As technological innovations advance, these techniques have been refined, which has not only improved the safety and effectiveness of procedures but also allowed nurses to play a more active role in managing complex conditions. This, in turn, fosters closer collaboration between nurses, physicians, and other healthcare professionals, enhancing the patient experience and creating a more holistic approach to treatment.

Furthermore, the introduction of new technologies and approaches enables nursing professionals to expand their competencies, facilitating the transition from traditional care to a more dynamic and multidisciplinary approach. Adequate education and the implementation of standardized protocols are crucial to ensure that these procedures are performed effectively and safely. By strengthening staff education in these techniques, the quality of care is improved, leading to more positive health outcomes^(1-2)^.
