# Supplementary material for: Invasive procedures in nursing: advancing treatment outcomes with innovative approaches
Source: Rev Lat Am Enfermagem. 2025 Jul 11;33:e4533. doi: 10.1590/1518-8345.0000.4533 (PMC12259219; doi:10.1590/1518-8345.0000.4533)
Supplement: Translation

# Translation: Procedimentos invasivos de enfermagem: avanço dos resultados do tratamento com abordagens inovadoras

**DOI:** 10.1590/1518-8345.0000.4534

**Authors:** Sara Mogedano-Cruz, Carlos Romero-Morales

**Affiliations:** 1 Universidad Europea de Madrid, Facultad de Medicina, Salud y Deporte, Madrid, Espanha.

As técnicas invasivas de enfermagem são procedimentos essenciais realizados em vários ambientes de saúde e desempenham um papel fundamental na evolução clínica dos pacientes. O escopo da prática dos enfermeiros varia de acordo com o país, e é importante entender as estruturas regulatórias e educacionais que moldam o papel dos enfermeiros na realização desses procedimentos^(1)^.

Na Espanha, os enfermeiros devem passar por educação e treinamento especializados para realizar essas intervenções com segurança. O treinamento geralmente inclui conhecimento teórico e experiência prática, que são adquiridos por meio de programas de enfermagem, cursos de pós-graduação e supervisão clínica. Esse treinamento garante que os enfermeiros estejam equipados com as habilidades necessárias para realizar procedimentos invasivos de forma eficaz e segura. Além da educação formal, os enfermeiros devem aderir a protocolos e diretrizes rigorosos para minimizar riscos e complicações.

As técnicas invasivas são definidas como procedimentos que envolvem a penetração no corpo para tratar doenças e melhorar o prognóstico e a qualidade de vida dos pacientes. No ambiente cada vez mais complexo do setor de saúde, em que os pacientes geralmente apresentam várias comorbidades, é fundamental que os profissionais de saúde recebam educação adequada para realizar essas intervenções com segurança e eficácia. Embora a frequência desses procedimentos possa variar de acordo com o contexto clínico, muitas dessas técnicas são rotineiras em hospitais e são realizadas diariamente^(2)^.

A relevância dessas técnicas está em sua capacidade de fornecer acesso a áreas internas do corpo, facilitando a administração dos tratamentos necessários. Essas intervenções não são apenas essenciais para o tratamento intensivo, mas também desempenham um papel importante no gerenciamento de várias condições de longo prazo. A execução adequada dessas técnicas pode melhorar significativamente os resultados dos pacientes, reduzir a probabilidade de complicações e afetar positivamente a qualidade de vida geral^(2-3)^.

Uma das técnicas invasivas mais comuns na enfermagem é a canulação do acesso venoso, um procedimento usado para a administração de fluidos e medicamentos, especialmente em situações de emergência. Além disso, nos últimos anos, a canulação de acesso central periférico ganhou relevância no tratamento de pacientes que necessitam de tratamentos intravenosos prolongados. Essa técnica permite a administração de medicamentos e fluidos, evitando danos às veias periféricas. O uso dessas opções terapêuticas resultou em um impacto positivo na permanência hospitalar e na satisfação do paciente^(3)^.

O cateterismo da bexiga é outra técnica fundamental usada para drenar a urina da bexiga em pacientes com retenção urinária, ou para monitorar com precisão a função renal. Apesar de ser um procedimento de rotina, ele apresenta riscos significativos, como infecções do trato urinário. Portanto, a implementação de protocolos específicos de inserção e manutenção tem se mostrado eficaz na redução da incidência de efeitos adversos^(4)^.

A estimulação de nervos periféricos foi recentemente integrada como uma abordagem complementar no tratamento da dor, oferecendo uma estratégia inovadora para a dor intensa e crônica. Essa técnica não só ajuda a diminuir a intensidade da dor, mas também permite uma avaliação mais precisa da função nervosa. Pesquisas sugerem que esse tipo de estimulação pode ser eficaz no tratamento de várias condições dolorosas, melhorando assim a qualidade de vida dos pacientes^(5)^.

A punção lombar é outro procedimento relevante usado para obter líquido cefalorraquidiano para fins diagnósticos ou terapêuticos. Essa intervenção requer um profundo entendimento anatômico e habilidades específicas para minimizar o risco de complicações^(1)^. Por fim, a drenagem do abscesso é crucial no tratamento de infecções localizadas, impedindo a progressão da infecção e evitando complicações como a sepse. A educação adequada para a realização desses procedimentos deve abranger tanto os aspectos técnicos quanto o manejo asséptico, pois o manuseio inadequado pode aumentar o risco de complicações adicionais^(2-4)^.

O entendimento completo e a execução correta dessas técnicas não apenas aprimoram o atendimento imediato ao paciente, mas também afetam o gerenciamento de longo prazo de várias condições. A educação continuada da equipe de saúde é essencial para garantir a segurança e a eficácia desses procedimentos, bem como para minimizar as complicações e melhorar os resultados clínicos. A educação deve ser um processo contínuo que se adapte às necessidades de mudança do ambiente clínico e aos avanços na prática da saúde^(1-2)^.

Além disso, uma abordagem multidisciplinar no setor de saúde destaca a importância da colaboração entre diferentes profissionais. Os enfermeiros, como prestadores de cuidados primários, devem trabalhar em conjunto com médicos, fisioterapeutas e outros especialistas para oferecer cuidados abrangentes que atendam às necessidades físicas, emocionais e sociais dos pacientes. Essa colaboração não apenas melhora a qualidade do atendimento, mas também contribui para criar uma experiência mais positiva para o paciente, facilitando uma recuperação mais rápida^(1)^.

É importante observar que a lista de procedimentos invasivos discutida neste artigo não é exaustiva, pois há muitos outros procedimentos realizados por enfermeiros, dependendo do contexto clínico. O escopo da prática dos enfermeiros varia internacionalmente, e é essencial reconhecer essas diferenças ao discutir procedimentos invasivos na prática de enfermagem.

Em conclusão, as técnicas de tratamento invasivo são componentes indispensáveis na prática da enfermagem e no atendimento ao paciente. Com o avanço das inovações tecnológicas, essas técnicas foram aprimoradas, o que não apenas melhorou a segurança e a eficácia dos procedimentos, mas também permitiu que os enfermeiros desempenhassem uma função mais ativa no gerenciamento de condições complexas. Isso, por sua vez, promove uma colaboração mais próxima entre enfermeiros, médicos e outros profissionais de saúde, aprimorando a experiência do paciente e criando uma abordagem mais holística para o tratamento.

Além disso, a introdução de novas tecnologias e abordagens permite que os profissionais de enfermagem ampliem suas competências, facilitando a transição do atendimento tradicional para uma abordagem mais dinâmica e multidisciplinar. A educação adequada e a implementação de protocolos padronizados são fundamentais para garantir que esses procedimentos sejam realizados de forma eficaz e segura. Ao reforçar o treinamento da equipe nessas técnicas, a qualidade do atendimento é aprimorada, levando a resultados de saúde mais positivos^(1-2)^.

# Translation: Procedimientos invasivos en enfermería: avanzando en los resultados del tratamiento con enfoques innovadores

**DOI:** 10.1590/1518-8345.0000.4532

**Authors:** Sara Mogedano-Cruz, Carlos Romero-Morales

**Affiliations:** 1 Universidad Europea de Madrid, Facultad de Medicina, Salud y Deporte, Madrid, España.

Las técnicas invasivas en enfermería son procedimientos esenciales que se realizan en diversos entornos de atención médica y desempeñan un papel fundamental en la mejora de los resultados de los pacientes. El alcance de la práctica de los enfermeros varía según el país, y es importante comprender los marcos regulatorios y educativos que dan forma al papel de los enfermeros en la realización de estos procedimientos^(1)^.

En España, los enfermeros deben someterse a una educación y formación especializada para realizar estas intervenciones de forma segura. La capacitación a menudo incluye tanto conocimientos teóricos como experiencia práctica, que se adquiere a través de programas de enfermería, cursos de postgrado y supervisión clínica. Esta capacitación garantiza que los enfermeros estén equipados con las habilidades necesarias para realizar procedimientos invasivos de manera efectiva y segura. Además de la educación formal, los enfermeros deben cumplir con estrictos protocolos y pautas para minimizar los riesgos y complicaciones.

Las técnicas invasivas se definen como procedimientos que implican la penetración en el organismo con el fin de tratar enfermedades y mejorar el pronóstico y la calidad de vida de los pacientes. En el entorno de atención médica cada vez más complejo, donde los pacientes a menudo presentan múltiples comorbilidades, es fundamental que los profesionales de la salud reciban la educación adecuada para realizar estas intervenciones de manera segura y efectiva. Si bien la frecuencia de dichos procedimientos puede variar según el contexto clínico, muchas de estas técnicas son rutinarias en los hospitales y se realizan a diario^(2)^.

La relevancia de estas técnicas radica en su capacidad para proporcionar acceso a áreas internas del cuerpo, facilitando la administración de los tratamientos necesarios. Estas intervenciones no solo son esenciales para la atención aguda, sino que también desempeñan un papel importante en el manejo de varias afecciones a largo plazo. La ejecución adecuada de estas técnicas puede mejorar significativamente los resultados de los pacientes, reducir la probabilidad de complicaciones y afectar positivamente la calidad de vida en general^(2-3)^.

Una de las técnicas invasivas más comunes en enfermería es la canulación del acceso venoso, un procedimiento utilizado para la administración de líquidos y medicamentos, especialmente en situaciones de emergencia. Además, en los últimos años, la canulación periférica de acceso central ha ganado relevancia en el manejo de pacientes que requieren tratamientos intravenosos prolongados. Esta técnica permite la administración de medicamentos y líquidos evitando daños en las venas periféricas. El uso de estas opciones terapéuticas ha tenido un impacto positivo en la estancia hospitalaria y la satisfacción del paciente^(3)^.

El cateterismo vesical es otra técnica fundamental utilizada para drenar la orina de la vejiga en pacientes con retención urinaria o para controlar con precisión la función renal. Aunque es un procedimiento de rutina, conlleva riesgos significativos, como infecciones del tracto urinario. Por lo tanto, la implementación de protocolos específicos de inserción y mantenimiento ha demostrado ser eficaz para reducir la incidencia de efectos adversos^(4)^.

La estimulación de los nervios periféricos se ha integrado recientemente como un enfoque complementario en el manejo del dolor, proporcionando una estrategia innovadora tanto para el dolor agudo como para el crónico. Esta técnica no solo ayuda a disminuir la intensidad del dolor, sino que también permite una evaluación más precisa de la función nerviosa. La investigación sugiere que este tipo de estimulación puede ser eficaz en el tratamiento de diversas afecciones dolorosas, mejorando así la calidad de vida de los pacientes^(5)^.

La punción lumbar es otro procedimiento relevante utilizado para obtener líquido cefalorraquídeo con fines diagnósticos o terapéuticos. Esta intervención requiere un profundo conocimiento anatómico y habilidades específicas para minimizar el riesgo de complicaciones^(1)^. Finalmente, el drenaje de abscesos es crucial para tratar infecciones localizadas, prevenir la progresión de la infección y evitar complicaciones como la sepsis. La educación adecuada para realizar estos procedimientos debe abarcar tanto los aspectos técnicos como el manejo aséptico, ya que un manejo inadecuado puede aumentar el riesgo de complicaciones adicionales^(2-4)^.

Un conocimiento profundo y una ejecución correcta de estas técnicas no solo mejoran la atención inmediata del paciente, sino que también afectan el manejo a largo plazo de diversas afecciones. La educación continuada del personal de salud es esencial para garantizar la seguridad y efectividad de estos procedimientos, así como para minimizar las complicaciones y mejorar los resultados clínicos. La educación debe ser un proceso continuo que se adapte a las necesidades cambiantes del entorno clínico y los avances en la práctica de la atención médica^(1-2)^.

Además, un enfoque multidisciplinario de la atención médica resalta la importancia de la colaboración entre diferentes profesionales. Los enfermeros, como proveedores de atención primaria, deben trabajar en conjunto con médicos, fisioterapeutas y otros especialistas para ofrecer una atención integral que aborde las necesidades físicas, emocionales y sociales de los pacientes. Esta colaboración no solo mejora la calidad de la atención, sino que también contribuye a crear una experiencia más positiva para el paciente, facilitando una recuperación más rápida^(1)^.

Es importante señalar que la lista de procedimientos invasivos discutidos en este artículo no es exhaustiva, ya que hay muchos otros procedimientos realizados por los enfermeros según el contexto clínico. El alcance de la práctica de los enfermeros varía a nivel internacional, y es esencial reconocer estas diferencias cuando se discuten procedimientos invasivos en la práctica de enfermería.

En conclusión, las técnicas invasivas de tratamiento son componentes indispensables en la práctica de enfermería y la atención al paciente. A medida que avanzan las innovaciones tecnológicas, estas técnicas se han ido perfeccionando, lo que no solo ha mejorado la seguridad y efectividad de los procedimientos, sino que también ha permitido a los enfermeros desempeñar un papel más activo en el manejo de afecciones complejas. Esto, a su vez, fomenta una colaboración más estrecha entre enfermeros, médicos y otros profesionales de la salud, mejorando la experiencia del paciente y creando un enfoque más holístico del tratamiento.

Además, la introducción de nuevas tecnologías y enfoques les permite a los profesionales de enfermería ampliar sus competencias, facilitando la transición del cuidado tradicional a un enfoque más dinámico y multidisciplinario. La educación adecuada y la implementación de protocolos estandarizados son cruciales para garantizar que estos procedimientos se realicen de manera efectiva y segura. Al fortalecer la educación del personal en estas técnicas, se mejora la calidad de la atención, lo que lleva a resultados de salud más positivos^(1-2)^.
